# Attachment, Emotion, and Physiological Coregulation Among Elderly Mothers and Their Adult Daughters

**DOI:** 10.1093/geroni/igab046.976

**Published:** 2021-12-17

**Authors:** Elizabeth Jain, Gisela Labouvie-Vief, Mark Lumley

**Affiliations:** 1 Aurora University, Saint Charles, Illinois, United States; 2 University of Geneva, University of Geneva, Geneve, Switzerland; 3 Wayne State University, Wayne State University, Michigan, United States

## Abstract

Examination of physiological coregulation among marital partners suggests a dynamic interplay between partner physiology. Further, attachment dimensions of anxiety and avoidance mediate this coregulation during conflict. This study examined the role of attachment and race in predicting physiological coregulation for mothers and their adult daughters during emotional discussions. A sample of 23 African American and 17 Caucasian mother-daughter pairs (aged 26 to 83) completed interview sessions and Relationships Questionnaires. Pairs engaged in discussions (neutral, conflict, happy), while monitoring heart rate. HR difference scores were computed between pairs (bps; 0 meant no difference). Multiple Regressions revealed attachment anxiety and avoidance predicted HR variation between pairs for the neutral and happy discussions, differently by racial group (F(7,33)=3.297, p < 0.01). For African American women, increased anxiety predicted increased HR variation during neutral and happy discussions, whereas for Caucasian women, increased avoidance predicted increased HR variation. However, during conflict anxiety singularly predicted increased HR covariation (b = 5.03, p = 0.01), for both groups. Low anxiety and low avoidance predicted physiological coregulation (lower HR variance between pairs). Increased anxious attachment predicted partner dysregulation (increased HR variation between pairs) across all 3 discussions, moderated by avoidance for the Caucasian women. Results suggest attachment plays a role in regulating physiology under emotional stress, and that there may be important cultural differences in this relationship. Further examination will explore the dynamic interplay between attachment and physiological coregulation across adulthood and later life.

